# Anti-PD-1 and Novel Combinations in the Treatment of Melanoma—An Update

**DOI:** 10.3390/jcm9010223

**Published:** 2020-01-14

**Authors:** Frank Friedrich Gellrich, Marc Schmitz, Stefan Beissert, Friedegund Meier

**Affiliations:** 1 Department of Dermatology, University Hospital Carl Gustav Carus, TU Dresden, 01307 Dresden, Germany; Stefan.Beissert@uniklinikum-dresden.de (S.B.);; 2 Skin Cancer Center at the University Cancer Centre Dresden and National Center for Tumor Diseases, 01307 Dresden, Germany; 3 Institute of Immunology, Medical Faculty Carl Gustav Carus, TU Dresden, Fetscherstraße 74, 01307 Dresden, Germany; mschmitz@mail.zih.tu-dresden.de; 4 National Centre for Tumor Diseases, University Hospital Carl Gustav Carus, TU Dresden, Fetscherstraße 74, 01307 Dresden, Germany

**Keywords:** melanoma, PD-1, PD-L1, novel combinations, combination therapies, BEMPEG, T-VEC, LAG-3, IDO1, uveal mealnoma, mucosal melanoma, acral melanoma, desmoplastic melanoma, brain metastases, adjuvant therapy, neoadjuvant therapy

## Abstract

Until recently, distant metastatic melanoma was considered refractory to systemic therapy. A better understanding of the interactions between tumors and the immune system and the mechanisms of regulation of T-cells led to the development of immune checkpoint inhibitors. This review summarizes the current novel data on the treatment of metastatic melanoma with anti-programmed cell death protein 1 (PD-1) antibodies and anti-PD-1-based combination regimens, including clinical trials presented at major conference meetings. Immune checkpoint inhibitors, in particular anti-PD-1 antibodies such as pembrolizumab and nivolumab and the combination of nivolumab with the anti-cytotoxic T-lymphocyte-associated protein 4 (CTLA-4) antibody ipilimumab can achieve long-term survival for patients with metastatic melanoma. The anti-PD-1 antibodies nivolumab and pembrolizumab were also approved for adjuvant treatment of patients with resected metastatic melanoma. Anti-PD-1 antibodies appear to be well tolerated, and toxicity is manageable. Nivolumab combined with ipilimumab achieves a 5 year survival rate of more than 50% but at a cost of high toxicity. Ongoing clinical trials investigate novel immunotherapy combinations and strategies (e.g., Talimogene laherparepvec (T-VEC), Bempegaldesleukin (BEMPEG), incorporation or sequencing of targeted therapy, incorporation or sequencing of radiotherapy), and focus on poor prognosis groups (e.g., high tumor burden/LDH levels, anti-PD-1 refractory melanoma, and brain metastases).

## 1. Introduction

Until recently, distant metastatic melanoma was considered refractory to systemic therapy. Patients with metastatic melanoma had a median overall survival of 6–10 months. A better understanding of the genetic alterations in melanoma cells and the interactions between tumors and the immune system led to the development of immune checkpoint inhibitors (CPIs). The United States Food and Drug Administration (FDA) approval of the anti-cytotoxic T-lymphocyte-associated protein 4 (CTLA-4) antibody ipilimumab in 2011 and the anti-programmed cell death protein 1 (PD-1) antibodies nivolumab and pembrolizumab in 2014 has radically changed the systemic treatment of metastatic melanoma and significantly improved its clinical outcome. The second breakthrough in the systemic therapy of metastatic melanoma was the targeted therapy with BRAF and MEK inhibitors that may be used if tumor cells harbor a BRAF-V600 mutation. This review summarizes the current novel data on the treatment of metastatic melanoma with anti-PD-1 antibodies and combinations with other treatment modalities, including clinical trials presented at major conference meetings.

## 2. Cutaneous Melanoma

### 2.1. Anti-PD-1 Antibodies

**Figure 1:**
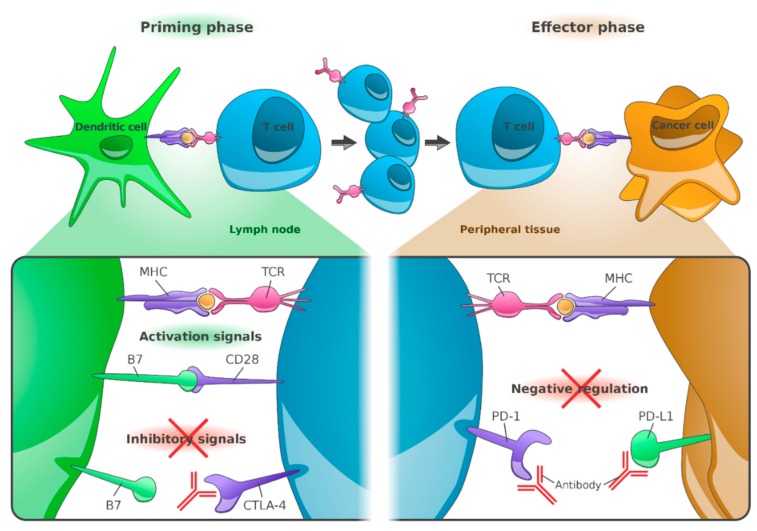
Anti-cytotoxic T-lymphocyte-associated protein 4 (CTLA-4), anti-programmed cell death protein 1 (PD-1) and anti-programmed death ligand 1 (PD-L1) antibodies: Mode of action. Peptides derived from tumor-associated antigens (TAA) are presented by the major histocompatibility complex (MHC) on the surface of cancer cells or DCs and recognized by T cells via their T-cell receptor (TCR). The co-stimulatory molecules B7-1 (or CD80) and B7-2 (or CD86), which are required for T-cell priming, provide an additional signal. T-cell activation leads to upregulation of CTLA-4 on T cells. Binding of CTLA-4 to B7 receptors of dendritic cells results in inhibition of T-cell activation. Anti-CTLA-4 blocking antibodies restore T-cell stimulation in the lymph nodes. Following long-term stimulation, the PD-1 receptor is upregulated by T cells. PD-L1 expressed on cancer cells binds to PD-1 receptors on T cells, which leads to their inhibition. PD-1/PD-L1 antibodies enhance the functional properties of effector T cells at the tumor site. (Reference: Figure adapted from Ribas A [1] and created by F.F. Gellrich).

The PD-1 receptor inhibits T-cell activity by interacting with its ligands: PD-L1 on tumor cells and antigen presenting cells, and PD-L2 on tumor cells, activated monocytes and dendritic cells (DC). This leads to an immunosuppressive tumor environment. The therapeutic use of blocking anti-PD-1 antibodies or anti-PD-L1 antibodies interferes with these immunosuppressive effects and strengthens the T-cell response to the tumor [1] (Figure 1). 

Nivolumab was the first anti-PD-1 antibody approved for the treatment of melanoma by the FDA in 2014. The approval was based on the results of the phase III study CheckMate 066 [2]. In total, 418 previously untreated patients with metastatic melanoma and wild-type BRAF received either nivolumab or dacarbazine. Nivolumab compared with dacarbazine achieved objective response rates of 40% vs. 13.9%, median progression-free survival rates of 5.1 months vs. 2.2 months, and 1 year survival rates of 72.9% vs. 42.1%. National Cancer Institute common terminology criteria for adverse events (CTCAE) grade 3 or 4 drug-related adverse events occurred in 11.7% of patients treated with nivolumab. An update of the phase I study on nivolumab in patients with metastatic melanoma showed a 5 year survival rate of 34% [3] (Table 1).

Pembrolizumab is another anti-PD-1 antibody approved by the FDA in 2014. The studies conducted to date showed comparable results in terms of both efficacy and toxicity [4,15]. KEYNOTE-001 enrolled 655 patients with melanoma. Previously treated or treatment-naive patients with metastatic melanoma received pembrolizumab 2 mg/kg every 3 weeks, 10 mg/kg every 3 weeks, or 10 mg/kg every 2 weeks. The overall response rate (ORR) was 52% with 25% complete remissions (CR), and the disease control rate (DCR) was 72%. The median PFS was 8.3 months in all patients and 16.9 months in treatment-naive patients, and the estimated 5 year overall survival (OS) rate was 34% and 41%, respectively. Treatment-related adverse events (TRAEs) occurred in 86% of patients, including 17% CTCAE grade 3 or 4 TRAEs. TRAEs led to study discontinuation in 7.8% of patients [5] (Table 1).

In conclusion, the anti-PD-1 antibodies nivolumab and pembrolizumab achieved an ORR of 40% to 50% and 5 year OS rates of 30 to 40% in patients with metastatic melanoma. Responses are durable. Toxicity is manageable. Most adverse events are immune-mediated adverse reactions such as endocrinopathies, pneumonitis, colitis, nephritis, and hepatitis [16].

### 2.2. Combination of Anti-PD-1 Antibodies with Anti-CTLA-4 Antibodies

The combined blockade of PD-1 and CTLA-4 enables inactivated tumor-specific T cells to multiply again and perform their effector function. This leads to immune activation of the so far immunosuppressed tumor microenvironment [17] (Figure 1). 

In the phase III CheckMate 067 trial [6], 945 therapy-naive patients with metastatic melanoma received the anti-PD-1 antibody nivolumab in combination with the anti-CTLA-4 antibody ipilimumab or nivolumab, or ipilimumab as monotherapy. This study was insufficiently powered in terms of a reliable comparative statistical evaluation of nivolumab plus ipilimumab vs. nivolumab. Objective response rates were 58% for the combination vs. 45% for nivolumab vs. 19% for ipilimumab. The combination of nivolumab with ipilimumab achieved a 5 year survival rate of 52% vs. 44% for nivolumab monotherapy vs. 26% for ipilimumab monotherapy. The most common adverse events were colitis and hepatitis under treatment with nivolumab +/− ipilimumab and exanthema, colitis and hypophysitis under ipilimumab monotherapy. CTCAE grade 3 and 4 adverse events were observed in 59%, 23% and 28% of the patients in the nivolumab plus ipilimumab, nivolumab, and ipilimumab group, respectively. Most CTCAE grade 3 and 4 adverse events improved under adequate immunosuppressive therapy [18]. The median time to resolution of adverse events was less than 12 weeks, with the exception of some events that had not yet resolved (e.g., endocrine events). No unknown long-term toxicity was reported (Table 1).

In the KEYNOTE-029 study, the anti-PD-1 antibody pembrolizumab was combined with ipilimumab. In total, 72% of patients received all 4 doses of pembrolizumab 2 mg/kg plus ipilimumab 1 mg/kg. 42% of patients remained on pembrolizumab monotherapy. In total, 61% of patients achieved an objective response. Estimated 1 year PFS was 69% and estimated 1 year OS was 89% [7]. In total, 60% of patients developed therapy-related adverse events and CTCAE grade 3 and 4 adverse events were observed in 27% of patients. Most common adverse events were hypothyroidism (16%) and hyperthyroidism (11%). No treatment-related deaths occurred.

In conclusion, the combination of nivolumab and ipilimumab appears to be superior to anti-PD-1 monotherapy in the treatment of metastatic melanoma. However, higher response rates (58% vs. 44%) and better 4 year survival rates (53% vs. 46%) are at the expense of higher toxicity (grade 3 and 4: 55% vs. 16%). The combination of pembrolizumab with low-dose ipilimumab achieved comparable high response rates with lower toxicity. However, this combination is not yet approved.

### 2.3. Duration of Therapy

In the 4 year update of the CheckMate 067 trial, the data of 103 patients who completed 2 years of pembrolizumab were analyzed [19]. The median follow-up after end of treatment was 20.3 months. Complete remission was achieved by 27.2% of patients, partial remission (PR) by 63.1% and stable disease (SD) by 9.7%. 18 months after completion of therapy, and 95.8% of patients with CR, 91.3% of patients with PR and 66.7% of patients with SD were still progression free.

In summary, anti-PD-1 antibodies show a durable antitumor activity in patients who have completed 2 years of therapy. However, further studies are required to determine the optimal therapy duration. 

### 2.4. Combination of Targeted Therapy with Anti-PD-1 Antibodies

Targeted therapy, i.e., BRAF inhibitors combined with MEK inhibitors, induce rapid responses and high response rates in patients with metastatic melanoma in the presence of a BRAF-V600 mutation [20]. However, in the majority of patients, the duration of response is limited due to acquired resistance [21]. By contrast, CPIs are slower-acting and induce less frequent but durable responses. There is growing evidence that BRAF/MEK inhibitors target multiple events in the cancer-immunity cycle. For example BRAF/MEK inhibitors promote the release of cancer cell antigens, cancer antigen presentation, infiltration of T cells into tumors, recognition of cancer cells by T cells and killing of cancer cells [22]. Combining targeted with immune therapy may achieve rapid responses, high response rates and durable responses with prolonged survival.

A phase III study (NCT02967692) is investigating the safety and efficacy of the anti-PD-1 antibody spartalizumab in combination with the BRAF inhibitor dabrafenib and the MEK inhibitor trametinib in untreated patients with BRAF V600-mutant metastatic melanoma. The data of 36 patients enrolled in part 1 (safety run-in cohort) and part 2 (biomarker cohort) of this study were presented at the 2019 ASCO Annual Meeting [8]. Treatment with spartalizumab combined with dabrafenib and trametinib achieved an ORR of 75% and a complete response rate of 42%. The 1 year OS rate was 86%, and the median OS was not reached. Toxicity appears to be high. CTCAE grade ≥ 3 adverse events occurred in 78% of patients, and adverse events leading to discontinuation of all three study drugs occurred in 17% patients. Adverse events included pyrexia, chills, fatigue, cough, and arthralgia [8]. The global part 3 of this study, which compares spartalizumab in combination with dabrafenib and trametinib with dabrafenib and trametinib alone, is ongoing (Table 1). 

In the KEYNOTE-022 phase-2 study (NCT02130466), 120 treatment-naive BRAF-V600E/K-mutant patients with advanced melanoma were randomized to receive the BRAF inhibitor dabrafenib and the MEK inhibitor trametinib in combination with pembrolizumab or placebo [9]. ORR was 63.3% in the triplet group compared with 71.7% in the doublet group. The reason for this difference was considered to be an imbalance in baseline patient characteristics with better prognostic factors in the doublet arm. PFS was 16.0 months in the triplet group vs. 10.3 months in the doublet group. However, the planned benefit for a statistically significant improvement was not reached. The 1 year OS rate was 79.9% and 72.9%, respectively (Table 1). After end of study treatment, a greater proportion of patients in the doublet arm (48.3%) received post-progression immunotherapy than in the triplet arm (15.0%). Grade 3–5 treatment-related adverse events occurred in 58.3% in the triplet arm and 26.7% in the doublet arm. The most common adverse events were fever, hepatitis and rash.

In phase 1 of the KEYNOTE-022 trial (NCT02130466), 15 BRAF-V600E/K-mutant patients were enrolled for triplet therapy. ORR was 73% [23]. Remarkably, 40% of the patients continued to respond at a median follow-up of 27 months, while the median duration of response was approximately 1 year for doublet therapy with BRAF and MEK inhibitors [24]. 

In conclusion, the efficacy of the anti-PD-1 antibodies spartalizumab or pembrolizumab combined with the BRAF inhibitor dabrafenib and the MEK inhibitor trametinib may exceed the efficacy of dabrafenib and trametinib alone. Toxicity appears to be high, but manageable. 

### 2.5. Combination of Anti-PD-1 Antibodies with Pegylated Engineered Interleukin-2 

**Figure 2:**
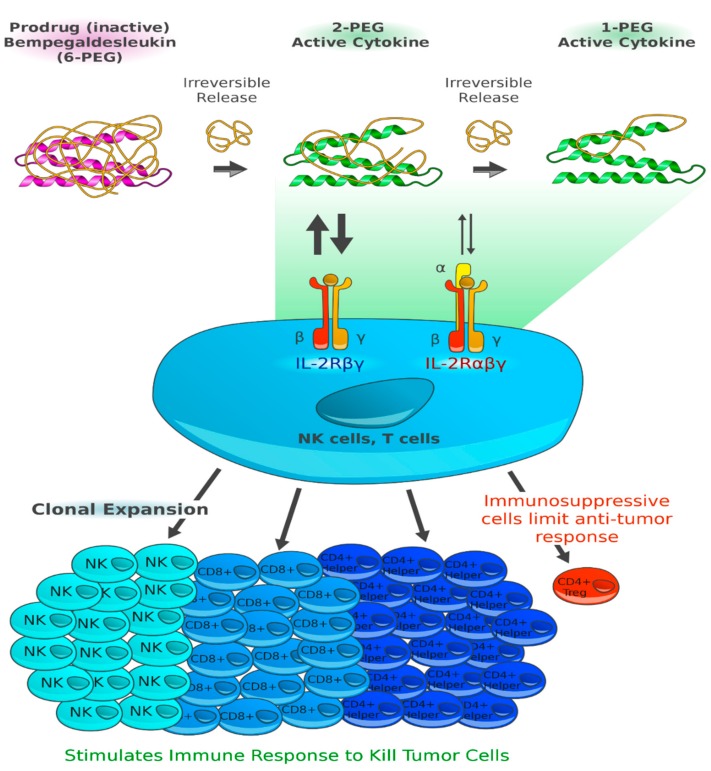
Bempegaldesleukin expands and activates cluster of differentiation (CD)8+ effector T cells and natural killer cells over regulatory T cells. Bempegaldesleukin (BEMPEG; NKTR-214) is a human recombinant inteleukin-2 (IL-2) attached to six releasable polyethylene glycol (PEG) chains that alter its pharmacokinetics and its receptor binding. When fully PEGylated, NKTR-214 is a prodrug with no biological activity. In vivo, the PEG chains slowly release to generate active IL-2 conjugates with limited binding to the IL-2Rα subunit, thereby favoring the dimeric βγ-IL-2 receptor (IL-2Rβγ; CD122). Consequently, NKTR-214 is selectively stimulating CD8+ T cells and natural killer (NK) cells over the undesirable T regulatory cells (Treg) [25]. (Reference: Figure adapted from Charych D [26] and created by F.F. Gellrich).

Bempegaldesleukin (BEMPEG; NKTR-214) is a prodrug of conjugated interleukin (IL)-2. The IL-2 core is conjugated to six releasable polyethylene glycol (PEG) chains. In vivo, the PEG chains slowly release to generate active IL-2 conjugates, selectively stimulating CD8+ T cells over regulatory T cells (Figure 2). BEMPEG increases tumor-infiltrating lymphocytes, T-cell clonality and PD-1 expression [25]. In an ongoing phase I/II study, the combination of BEMPEG with the anti-PD-1 antibody nivolumab in patients with previously untreated metastatic melanoma is being investigated. At the 2019 ASCO Annual Meeting, the data of 38 efficacy-evaluable patients were presented [10]. BEMPEG combined with nivolumab achieved an ORR of 53%, a CR in 34% of patients, and a DCR of 74%. The responses were durable with 80% ongoing responses after a median follow-up of 12.7 months. Biomarker analyses of baseline tumor biopsies identified immune signatures that accumulate for treatment response, e.g., interferon (IFN)γ or CD8+ tumor-infiltrating lymphocytes (TIL). Notably, responses were even observed in patients with the least favorable tumor microenvironment. The most common adverse events were flu-like symptoms, rash and fatigue. Therapy had to be discontinued by 9.8% of patients due to TRAEs [10] (Table 1).

In conclusion, the biomarker analyses identified baseline immune signatures that correlated with response to BEMPEG combined with nivolumab. Responses were seen in both favorable and unfavorable tumor microenvironments.

### 2.6. Combination of Anti-PD-1 Antibodies with T-VEC

**Figure 3:**
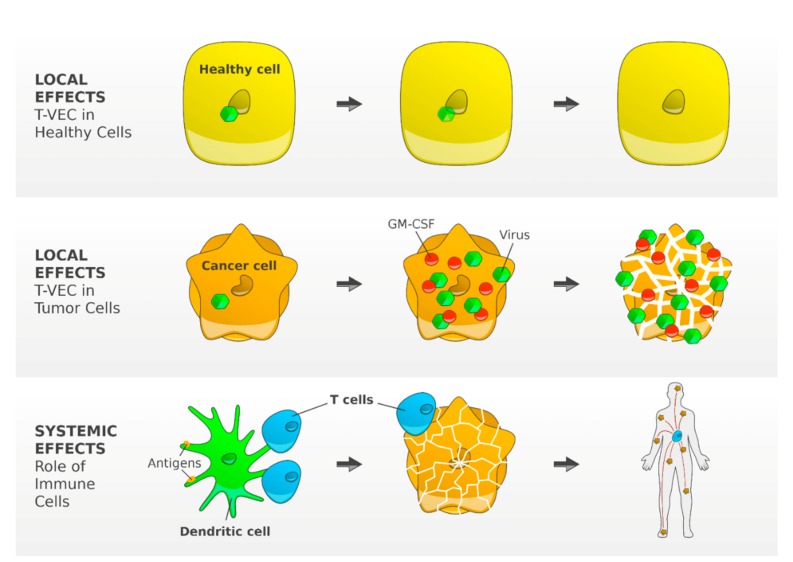
T-VEC—mechanism of action. T-VEC is a type 1 herpes simplex virus genetically modified by the deletion of two non-essential viral genes. The functional deletion of the gene herpesvirus neurovirulence factor (ICP34.5) attenuates viral pathogenicity and enhances tumor-selective replication. Inside a normal cell, the virus is unable to replicate. In tumor cells, the virus replicates and induces granulocyte-macrophage colony-stimulating factor (GM-CSF). The lysed tumor cells release viruses, GM-CSF and TAAs. GM-CSF attracts DCs that process and present TAAs to T cells resulting in an efficient induction and activation of tumor-reactive T cells [27]. (Reference: Figure adapted from Andtbacka R [27] and created by F.F. Gellrich).

Talimogene laherparepvec (T-VEC) is the first oncolytic virus therapy approved for patients with metastatic melanoma. T-VEC is a type 1 herpes simplex virus genetically modified to preferentially replicate in tumor cells. In tumor cells, the virus replicates and secretes Granulocyte-macrophage colony-stimulating factor (GM-CSF). The tumor cells lyse and release viruses, GM-CSF and tumor cell-associated antigens. GM-CSF attracts DCs that process and present tumor cell antigens to T cells. T cells are then programmed to identify and kill tumor cells [27] (Figure 3). Altogether, T-VEC creates an immunogenic tumor microenvironment and may enhance the efficacy of anti-PD-1 antibodies. In particular non-immunogenic tumors may benefit from a combination of T-VEC with CPIs [28].

In a phase Ib study, patients with metastatic melanoma and injectable lesions without prior systemic therapy were treated with T-VEC intralesionally and the anti-PD-1 antibody pembrolizumab. In total, 21 patients were enrolled, and the maximum treatment period was 2 years. At the 2018 Society for Melanoma Research Annual Meeting, the updated data of this study were presented. The median follow-up time was 36.8 months. Therapy was generally well tolerated, with fatigue, fever, and chills as the most common adverse events. The ORR was 67% with a complete response rate of 43% and 57% of patients remained in response [11,12]. Median progression-free survival (PFS) and OS were not reached at the data cutoff. The 36 month PFS and OS rates were 53.6% and 71%, respectively (Table 1). 

In this study, the combination of T-VEC and pembrolizumab was well tolerated and induced durable responses in the majority of patients. The randomized phase III study comparing T-VEC combined with pembrolizumab to pembrolizumab alone (NCT02263508) has completed enrollment and is currently ongoing [29]. 

### 2.7. Combination of Anti-PD-1 Antibodies with Anti-Lymphocyte Activation Gene (LAG)-3 Antibodies

Lymphocyte activation gene-3 (LAG-3) is an immune cell receptor that regulates a checkpoint pathway limiting the activity of T cells. Signaling via LAG-3 and other T-cell inhibitory receptors (e.g., PD-1) can lead to T-cell dysfunction and tumor immune escape. Simultaneous blockade of LAG-3 and PD-1 may synergistically restore T-cell activation and increase antitumor immunity [13].

In a phase 1/2a study (NCT01968109), the anti-LAG-3 antibody BMS-986016 in combination with the anti-PD-1 antibody nivolumab demonstrated peripheral T-cell activation, preliminary clinical activity and tolerability [30]. Patients progressed on prior anti-PD-1/PD-L1 therapy were treated with anti-LAG-3 antibody in combination with nivolumab. ORR was 16% and DCR was 45%. Grade 3/4 treatment-related adverse events occurred in 9% of patients [13] (Table 1).

In conclusion, the anti-LAG-3 antibody BMS-986016 showed efficacy in anti-PD-1/PD-L1-refractory patients, while toxicity is comparable to nivolumab monotherapy. 

### 2.8. Combination of Anti-PD-1 Antibodies with IDO1 Inhibitors

Tumors may evade immunosurveillance through upregulation of the enzyme indoleamine 2,3-dioxygenase 1 (IDO1). In the phase 1/2 ECHO-202/KEYNOTE-037 study, the IDO1 inhibitor epacadostat in combination with the anti-PD-1 antibody pembrolizumab was well tolerated and showed encouraging antitumor activity in multiple advanced tumors [31]. Based on this trial, 706 patients with unresectable stage III or IV melanoma were randomly assigned to receive pembrolizumab plus epacadostat or placebo [14]. In this phase 3 study (NCT02752074), epacadostat plus pembrolizumab did not improve PFS or OS compared with placebo plus pembrolizumab (Table 1). 

## 3. Adjuvant Therapy

There is a substantial risk of metastasis in resected high-risk melanoma. In patients with resected locoregional metastases, 1 year recurrence rates range from 8% to 76% and 10 year melanoma-specific survival rates range from 24% to 88%, depending on the tumor load [32,33,34]. For many years, no adjuvant therapy was available to be effective in reducing recurrence and mortality in resected high-risk melanoma. Recent developments in adjuvant therapy have changed the standard of care for these patients. 

In 2015, the FDA approved the anti-CTLA-4 antibody ipilimumab as an adjuvant therapy for patients with melanoma with regional lymph node metastases who have undergone complete resection. In a phase III trial, 951 patients were randomly assigned to ipilimumab 10 mg/kg or placebo every 3 weeks for four doses, then every 3 months for up to 3 years. The rate of OS at 5 years was 65.4% in the ipilimumab group, as compared with 54.4% in the placebo group. However, toxicity was high. CTCAE grade 3 or 4 immune-related adverse events occurred in 41.6% of patients in the ipilimumab group and in 2.7% of patients in the placebo group. In the ipilimumab group, 5 patients (1.1%) died due to immune-related adverse events [35,36].

In the phase III CheckMate 238 trial [37], patients who had undergone complete resection of locoregional or distant metastases were treated with anti-PD-1 nivolumab or anti-CTLA-4 ipilimumab for one year. The 1 year recurrence-free survival rates were 70.5% in the nivolumab arm and 60.8% in the ipilimumab arm. At a minimum follow-up of 24 months, recurrence-free survival rates continued to be higher for nivolumab vs. ipilimumab with 62.6% vs. 50.2% [38]. Toxicity was lower in the nivolumab arm with adverse events requiring therapy discontinuation in 9.7% of patients compared with 42.6% of patients in the ipilimumab arm. Altogether, adjuvant therapy with nivolumab achieved an improved recurrence-free survival and a lower rate of toxicity, compared with ipilimumab.

In the phase III EORTC 1325 study [39], patients with completely resected locoregional metastases received anti-PD-1 pembrolizumab (514 patients) or placebo (505 patients) for 1 year. At a median follow-up of 15 months, pembrolizumab was associated with significantly longer recurrence-free survival than placebo with a 1 year rate of recurrence-free survival of 75.4% vs. 61.0%. CTCAE grade 3–5 adverse events were reported in 14.7% of the patients in the pembrolizumab group and in 3.4% of patients in the placebo group. There was one treatment-related death due to myositis in the pembrolizumab group.

In summary, adjuvant CPI therapy with anti-CTLA-4 ipilimumab showed both a recurrence-free survival and overall survival benefit, but at a cost of high toxicity. Anti-PD-1 antibodies such as nivolumab and pembrolizumab, achieved higher recurrence-free survival rates with lower toxicity. Given the significant recurrence-free survival benefit, a significant overall survival benefit is expected. Nivolumab was approved for adjuvant treatment of patients who had undergone resection of melanoma and resection of all sites of disease. Pembrolizumab was approved for adjuvant treatment of patients who have melanoma with lymph node involvement who underwent complete resection.

## 4. Neoadjuvant Therapy

Besides adjuvant treatment, patients with regional lymph node metastases may also be treated in the neoadjuvant setting, before undergoing resection. Advantages may be the induction of a stronger immune response and the reduction of tumor burden. Treatment response may provide a predictive marker for additional adjuvant therapy. 

In a phase II trial investigating different neoadjuvant strategies, 86 patients were included [40]. In arm A, patients received 2 × ipilimumab 3 mg/kg plus nivolumab 1 mg/kg; in arm B, 2 × ipilimumab 1 mg/kg plus nivolumab 3 mg/kg; and in arm C, 2 × ipilimumab 3 mg/kg followed immediately by 2 × nivolumab 3 mg/kg. CTCAE grade ≥ 3 AEs occurred in 40%, 20%, and 50% in arm A, B and C, respectively. Arm C was closed due to high toxicity. Pathologic response rates were 80% and 77% in arm A and B with 43% and 57% complete responses, respectively. Pathologic response appeared to be the strongest marker for relapse-free survival. Altogether, ipilimumab 1 mg/kg plus nivolumab 3 mg/kg appeared to be the most attractive for further studies. 

In summary, CPIs have also been investigated in patients with palpable lymph node metastases in the neoadjuvant setting. These studies show promising results and further studies are expected in the near future.

## 5. Brain Metastases

Melanoma, along with lung and breast cancer, is one of the most common causes of brain metastases. The prognosis for melanoma patients with brain metastases is unfavorable with a median overall survival of approximately 4 months. The established local treatments include surgical resection, stereotactic radiosurgery and whole brain radiotherapy [41]. 

The approval of effective targeted and immune therapies has significantly improved the prognosis of metastatic melanoma including brain metastases with a median OS for patients with brain metastases of approximately 7 months for anti-CTLA-4 ipilimumab [42], 10 months for anti-PD-1 pembrolizumab or nivolumab [43] and up to 24 months for BRAF and MEK inhibitors [44]. Recently, combination therapy with anti-PD1 and anti-CTLA-4 showed unprecedented results [43,45]. 

### 5.1. Nivolumab Plus Ipilimumab

At the 2019 ASCO Annual Meeting, updated efficacy and safety results of the phase II study CheckMate 204 (NCT02320058) for patients with melanoma brain metastases (MBM) were reported [46]. In this phase II study, patients with MBM were enrolled into two cohorts: (cohort A) patients with asymptomatic brain metastases and no steroid treatment, and (cohort B) patients with symptomatic brain metastases with or without steroid treatment. All patients received nivolumab plus ipilimumab (4 times), then nivolumab until tumor progression or toxicity. In cohort A, the updated data of 101 asymptomatic MBM patients with a median follow-up of 20.6 months were reported. Intracranial and extracranial responses were largely concordant with an intracranial objective response rate of 54%. In total, 87% of patients showed an ongoing response. The overall survival rate at 18 months was 75%, the median OS has not been reached. In cohort B, new data of 20 patients with a median follow-up of 5.2 months were presented. The intracranial objective response rate dropped to 16.7%, and the median overall survival was 8.7 months. In both cohorts, more than 50% of patients experienced grade 3 to 4 adverse events that were comparable to patients without brain metastases [47]. 

In the ABC trial (NCT02374242), 76 patients with MBM were enrolled in three cohorts. Patients with asymptomatic MBM without prior local brain therapy were treated with nivolumab plus ipilimumab (cohort A) or nivolumab (cohort B). Previously treated patients, patients with neurological symptoms or leptomeningeal metastases were treated with nivolumab (cohort C). The 3 year overall survival rates were 49% (cohort A) vs. 42% (cohort B) vs. 19% (cohort C). Intracranial response rates were 51% (cohort A) vs. 20% (cohort B) vs. 6% (cohort C). Grade 3 or 4 treatment-related adverse events occurred in 54% patients of cohort A, 20% in cohort B and 13% in cohort C [43,48].

### 5.2. Radiotherapy Combined with Immune Checkpoint Inhibitors

Despite the encouraging study data, approximately half of patients with MBM fail to respond to systemic therapy. Response duration appears to be shorter than that in extracranial disease, emphasizing the need of combination or new treatment strategies. Several retrospective analyses suggest that combining stereotactic radiosurgery (SRS) with active systemic therapies improves melanoma brain metastases (MBM) control and prolongs survival without increasing toxicity [44,49]. Preclinical and clinical data suggest that radiotherapy can cause disruption of the blood-brain barrier enhancing drug delivery to the brain [50]. Moreover, the combination of radiotherapy and immune CPIs may increase the antitumor response by promoting antigen presentation and T-cell activation [51,52].

In a large real-life cohort of patients with MBM treated with CPIs or BRAF/MEK inhibitors, the risk of death was decreased by 40% for patients treated with radiotherapy, in comparison with those who did not receive radiotherapy [44]. In a retrospective study, data of 208 patients treated with SRS or whole brain radiation therapy in combination with CPIs or BRAF/MEK inhibitors within a 6-week interval to radiotherapy were analyzed [49]. The best survival was seen in patients treated with anti-PD-1 plus anti-CTLA-4 or anti-PD-1 alone combined with SRS with 12 month survival rates of 100% and 70%, respectively.

### 5.3. Immune Checkpoint Inhibitors in Melanoma Brain Metastases—Conclusions

In patients with asymptomatic MBM, the anti-PD-1 antibody nivolumab combined with the anti-CTLA-4 antibody ipilimumab achieved a high rate of durable intracranial responses supporting nivolumab plus ipilimumab as a first-line treatment in these patients. Patients with symptomatic MBM are challenging to treat. Some patients can benefit from nivolumab plus ipilimumab. Further studies in patients with symptomatic MBM are required to evaluate combination or new treatment strategies such as radiotherapy combined with CPIs or combinations of BRAF/MEK inhibitors with CPIs. Retrospective studies suggest that combining stereotactic radiosurgery with CPIs prolongs overall survival. 

## 6. Uveal Melanoma

Uveal melanoma (UM) is rare and accounts for approximately 5% of melanoma cases [53]. UM is characterized by mutations in GNAQ or GNA11 resulting in activation of the mitogen-activated protein kinase (MAPK) and other signaling pathways [54,55]. Approximately 40%–50% of patients with UM will develop metastatic disease. Metastatic UM is often fatal because the most common metastatic site is the liver [56]. There is currently no standard therapy for metastatic UM. Treatment options proposed in patients with metastatic UM are the same as those in patients with cutaneous melanoma. The prognosis of uveal melanoma is poor compared to cutaneous melanoma posing a particular treatment challenge. 

In a French retrospective study [57], 210 patients with metastatic UM were treated with the anti-CTLA-4 antibody ipilimumab or the anti-PD-1 antibodies nivolumab or pembrolizumab. No partial or complete response of metastases was observed. SD was seen in 32% of patients. OS of patients treated with immunotherapy was not significantly different from those of patients treated with chemotherapy, with a median OS of 13.38 months vs. 11.02 months. 

In a German retrospective study [58], 86 patients with metastatic UM were treated with anti-PD-1 antibodies with a response rate of 4.7% and a median OS of 14 months for pembrolizumab-treated and 10 months for nivolumab-treated patients. Fifteen patients were treated with anti-PD-1 antibodies combined with anti-CTLA-4 antibodies with a PR seen in two patients and a median OS not reached. 

In a phase II study, 35 patients with previously untreated or treated metastatic UM received anti-PD-1 nivolumab combined with anti-CTLA-4 ipilimumab [59]. A PR was seen in 17% of patients, and a stable disease in 53% of patients. Median OS was 1.6 years, and the 1 year OS rate was 62%. CTCAE grade 3 or 4 adverse events occurred in 40% of patients, 29% of patients had to discontinue therapy. 

In summary, single-agent CPI therapy in metastatic uveal melanoma has yielded poor results. Anti-PD-1 combined with anti-CTLA-4 has the potential to improve response rates and survival. However, toxicity is high.

## 7. Mucosal Melanoma

Mucosal melanoma is a rare subtype of melanoma, and occurs in the sinonasal cavity, oral cavity, anorectal region, or urogenital tract [60,61]. The prognosis of mucosal melanoma appears to be poorer than that of cutaneous melanoma, since mucosal melanoma is often diagnosed at an advanced stage and has particular clinical and genetic characteristics [62]. 

In a French multicenter retrospective study, 151 patients with metastatic mucosal melanoma received immunotherapy with anti-CTLA-4 (50.3%) or anti-PD-1 antibodies (49.7%). The objective response rate was 11.9%, and the DCR was 29.8%. The OS of mucosal melanoma patients treated with CPIs appeared to be longer than that of patients treated with chemotherapy, with a median OS of 15.97 months and 8.82 months, respectively. In conclusion, immunotherapy appears to improve overall survival for patients with metastatic mucosal melanoma [57]. In a pooled analysis [63], the outcome of patients with metastatic mucosal melanoma treated in trials either with single-agent nivolumab (*n* = 86) or the combination of nivolumab and ipilimumab (*n* = 35) was reported. For nivolumab monotherapy, the objective response rate was 23.3% in patients with mucosal melanoma, compared with 40.9% for patients with cutaneous melanoma. Median PFS was 3.0 months and 6.2 months for mucosal and cutaneous melanoma. Treatment with the combination of nivolumab and ipilimumab achieved an ORR of 37.1% in mucosal melanoma, compared with 60.4% seen in patients with cutaneous melanoma. Median PFS was 5.9 months and 11.7 months for mucosal and cutaneous melanoma. CTCAE grade 3 or 4 adverse events occurred in 8.1% of patients under treatment with nivolumab and in 40.0% of patients under treatment with nivolumab plus ipilimumab. 

### Mucosal Melanoma—Conclusions

Immunotherapy can be successful in mucosal melanoma. However, response rates are lower than in cutaneous melanoma. Anti-PD-1 nivolumab combined with anti-CTLA-4 ipilimumab appears to have greater efficacy than nivolumab alone. Clinical trials for patients with mucosal melanoma remain a key priority. 

## 8. Acral and Desmoplastic Melanoma

Acral lentiginous melanomas are considered a *distinct* subgroup with *unique* clinical, morphologic, and genetic characteristics [62], lower tumor mutational burden [64] and poorer prognosis than non-acral cutaneous melanomas [65]. In a retrospective analysis, 25 patients with acral melanoma received pembrolizumab or nivolumab [66]. ORR was 32%, median PFS 4.1 months and median OS 31.7 months, supporting the use of PD-1 blockade in clinical practice. 

Desmoplastic melanoma is characterized by a lack of actionable driver mutations and is highly associated with UV-induced DNA damage [67]. In a retrospective study, 60 patients with advanced desmoplastic melanoma treated with anti-PD-1 or anti-PD-L1 antibodies were identified [68]. ORR was 70% with 32% complete remissions. Patients with advanced desmoplastic melanoma appear to benefit from anti-PD-1/PD-L1 therapy. The benefit is expected to result from the high mutation burden. 

## 9. Immune-Related Adverse Events

Therapy with CPIs is associated with a wide spectrum of adverse events related to the mechanism of action. ICIs can induce immune-related adverse events (irAEs) in all organ systems, and most commonly affect the skin, gastrointestinal tract, lungs, and the endocrine, musculoskeletal, renal, nervous, hematologic, cardiovascular, and ocular systems. Severe irAEs occur in 10 to 20% of patients under monotherapy with nivolumab or pembrolizumab [2,5] and in more than 50% of patients under nivolumab combined with ipilimumab [69]. IrAEs may affect quality of life, may cause loss of organ function, and may even lead to death. Hence, toxicity of ICIs requires early detection and competent management, and patients and physicians should be aware that any symptoms may be treatment-related. The ASCO has developed guidelines on the management of irAEs [70]. General recommendations include: (1) Other causes should be excluded (e.g., infection, tumor progression). (2) For grade 2 toxicities corticosteroids may be administered. (3) For grade 3 toxicities, high-dose corticosteroids may be administered and subsequently tapered for at least 4 weeks. (4) If there is no improvement within 48 to 72 h, immunosuppressive therapy may be escalated (e.g., infliximab).

Notably, it was recently shown that treatment of mice with tumor necrosis factor (TNF) inhibitors concomitantly with anti-PD-1 and anti-CTLA-4 antibodies ameliorates immune-related colitis and, in addition, improves anti-tumor efficacy [71]. These data suggest that it is feasible to dissociate efficacy and toxicity of combined immune checkpoint blockade.

## 10. Conclusions

Impressive progress has been made in the treatment of patients with metastatic melanoma. Immune checkpoint inhibitors, in particular anti-PD-1 antibodies such as pembrolizumab and nivolumab and the combination of nivolumab with the anti-CTLA-4 antibody ipilimumab, can achieve long-term survival for patients with metastatic melanoma with 5 year survival rates of more than 40% and 50%, respectively. The anti-PD-1 antibodies nivolumab and pembrolizumab were also approved for adjuvant treatment of patients with resected metastatic melanoma.

Anti-PD-1 antibodies appear to be well tolerated, and toxicity is manageable. However, immune-related adverse events may be irreversible in rare cases. Patients treated with nivolumab combined with ipilimumab achieve a 5 year survival rate of 52% but at a cost of high toxicity with very rare cases of fatal outcome.

However, there are patients who do not benefit from treatment. These poor prognosis groups include patients with high tumor burden or high lactate dehydrogenase (LDH) levels, uveal melanoma, mucosal melanoma, brain metastases and anti-PD-1 refractory melanoma.

Ongoing clinical trials investigate novel immunotherapy combinations and strategies (e.g., T-VEC, BEMPEG, incorporation or sequencing of targeted therapy, and incorporation or sequencing of radiotherapy), and focus on poor prognosis groups (e.g., high tumor burden/LDH levels, anti-PD-1 refractory melanoma, and brain metastases).

## Figures and Tables

**Table 1:** Clinical Studies—Summary.

Clinical Trial	Phase	Status (January 2020)	Study Treatment	Number of Patients	ORR	OS	References
NCT00730639	1	Active, not recruiting	Nivolumab	418	40%	34% (5 year)	[2,3]
NCT01295827	1	Completed	Pembrolizumab	655	52%	34% (5 year)	[4,5]
NCT01844505	3	Active, not recruiting	Nivolumab + Ipilimumab	945	58%	52% (5 year)	[6]
NCT02089685	1/2	Active, not recruiting	Pembrolizumab + Ipilimumab	153	61%	89% (1 year)	[7]
NCT02967692	3	Recruiting	Spartalizumab + Dabrafenib + Trametinib	34	75%	86% (1 year)	[8]
NCT02130466	1/2	Active, not recruiting	Pembrolizumab + Dabrafenib + Trametinib	120	63%	79.9% (1 year)	[9]
NCT02983045	1/2	Recruiting	Nivolumab + BEMPEG (pegIL-2)	38	53%	N/A	[10]
NCT02263508	3	Active, not recruiting	Pembrolizumab + T-VEC	21	67%	71% (3 year)	[11,12]
NCT01968109	1/2	Recruiting	Nivolumab + BMS-986016 (anti-LAG-3)	43 anti-PD-1 refractory	16%	N/A	[13]
NCT02752074	3	Completed	Pembrolizumab + epacadostat (IDO1i)	706	34%	74% (1 year)not sig.	[14]

BEMPEG: Bempegaldesleukin; pegIL-2: pegylated interleukin 2; T-VEC: Talimogene laherparepvec; LAG-3: Lymphocyte activation gene-3; IDO1i: indoleamine 2,3-dioxygenase-1 inhibitor; ORR: overall response rate; OS: overall survival.

## Abbreviations

FDA	United States Food and Drug Administration
CTLA-4	cytotoxic T-lymphocyte-associated protein 4
PD-1	programmed cell death protein 1
ASCO	American Society of Clinical Oncology
CTCAE	criteria for adverse events
ORR	overall response rate
CR	complete remission
PR	partial remission
SD	stable disease
DCR	disease control rate
OS	overall survival
PFS	progression-free survival
TRAE	treatment related adverse event
irAE	immune-related adverse event
CPI	immune checkpoint inhibitor
IL-2	interleukin-2
PEG	polyethylene glycol
T-VEC	talimogene laherparepvec
GM-CSF	granulocyte-macrophage colony-stimulating factor
MBM	melanoma brain metastases
UM	uveal melanoma
MAPK	mitogen-activated protein kinase
MHC	major histocompatibility complex
TCR	T-cell receptor
TAA	tumor-associated antigens
DC	dendritic cell
NK	natural killer cells
Treg	T regulatory cells
